# CD8 Cells of Patients with Diffuse Cutaneous Leishmaniasis Display Functional Exhaustion: The Latter Is Reversed, *In Vitro,* by TLR2 Agonists

**DOI:** 10.1371/journal.pntd.0000871

**Published:** 2010-11-02

**Authors:** Joselín Hernández-Ruiz, Norma Salaiza-Suazo, Georgina Carrada, Sofía Escoto, Adriana Ruiz-Remigio, Yvonne Rosenstein, Alejandro Zentella, Ingeborg Becker

**Affiliations:** 1 Departamento de Medicina Experimental, Facultad de Medicina, Universidad Nacional Autónoma de México, Hospital General de México OD, México Distrito Federal, Mexico; 2 Secretaría de Salud del Estado de Tabasco y Universidad Juárez Autónoma de Tabasco, Villahermosa, Mexico; 3 Instituto de Biotecnología y Posgrado en Ciencias Bioquímicas, Universidad Nacional Autónoma de México, Cuernavaca, Mexico; 4 Departamento de Bioquímica, Instituto Nacional de Ciencias Médicas y Nutrición “Salvador Zubirán”, Secretaría de Salud, México Distrito Federal, Mexico; 5 Instituto de Investigaciones Biomédicas, Universidad Nacional Autónoma de México, México Distrito Federal, Mexico; 6 Dirección de Investigación, Hospital General de México OD, México Distrito Federal, Mexico; Institut Pasteur, France

## Abstract

*Leishmania mexicana* (Lm) causes localized (LCL) and diffuse (DCL) cutaneous leishmaniasis. DCL patients have a poor cellular immune response leading to chronicity. It has been proposed that CD8 T lymphocytes (CD8) play a crucial role in infection clearance, although the role of CD8 cytotoxicity in disease control has not been elucidated. Lesions of DCL patients have been shown to harbor low numbers of CD8, as compared to patients with LCL, and leishmanicidal treatment restores CD8 numbers. The marked response of CD8 towards *Leishmania* parasites led us to analyze possible functional differences between CD8 from patients with LCL and DCL. We compared IFNγ production, antigen-specific proliferation, and cytotoxicity of CD8 purified from PBMC against autologous macrophages (MO) infected with *Leishmania mexicana* (MOi). Additionally, we analyzed tissue biopsies from both groups of patients for evidence of cytotoxicity associated with apoptotic cells in the lesions. We found that CD8 cell of DCL patients exhibited low cytotoxicity, low antigen-specific proliferation and low IFNγ production when stimulated with MOi, as compared to LCL patients. Additionally, DCL patients had significantly less TUNEL^+^ cells in their lesions. These characteristics are similar to cellular “exhaustion” described in chronic infections. We intended to restore the functional capacity of CD8 cells of DCL patients by preincubating them with TLR2 agonists: Lm lipophosphoglycan (LPG) or Pam3Cys. Cytotoxicity against MOi, antigen-specific proliferation and IFNγ production were restored with both stimuli, whereas PD-1 (a molecule associated with cellular exhaustion) expression, was reduced. Our work suggests that CD8 response is associated with control of Lm infection in LCL patients and that chronic infection in DCL patients leads to a state of CD8 functional exhaustion, which could facilitate disease spread. This is the first report that shows the presence of functionally exhausted CD8 T lymphocytes in DCL patients and, additionally, that pre-stimulation with TLR2 ligands can restore the effector mechanisms of CD8 T lymphocytes from DCL patients against *Leishmania mexicana*-infected macrophages.

## Author Summary

*Leishmania mexicana* causes localized and diffuse cutaneous leishmaniasis. Whereas the former is a benign form the disease, diffuse cutaneous leishmaniasis is a chronic disfiguring disease, for which no cure is available, and the immune cells of these patients respond poorly to the parasite. It has been proposed that the elimination of *Leishmania*-infected cells by CD8 T cells is crucial for disease control. We compared the functional characteristics of CD8 T cells from patients with localized and diffuse cutaneous leishmaniasis. We found that CD8 T cells from patients with diffuse cutaneous leishmaniasis were functionally exhausted, as compared to patients with the benign form of the disease. We were able to restore functional capacity of these cells by culturing them with molecules that stimulate TLR2. This is the first report showing that stimulation of the TLR2 can restore effector mechanisms in functionally exhausted CD8 cells from patients with diffuse cutaneous leishmaniasis. This finding will help design novel treatment schemes for patients infected with the parasite *Leishmania mexicana* who have the progressive, incurable form of diffuse cutaneous leishmaniasis.

## Introduction

Leishmaniasis is a zoonotic disease that infects humans as well as a variety of mammalian species. Several *Leishmania* species, such as *L. mexicana*, *L. amazonensis*, *L. braziliensis* and *L. aethiopica* can cause two opposite clinical forms of cutaneous leishmaniasis: localized cutaneous leishmaniasis (LCL) and diffuse cutaneous leishmaniasis (DCL) [1]. While the former is relatively benign, consisting of a single ulcer that forms at the infection site, patients with DCL have a continuous uncontrolled spread of the parasite throughout the skin and, in advanced stages, these patients also show parasite invasion of the oro- and nasopharygeal mucosae. Although the prevalence of DCL patients in Mexico is low, they represent a public health problem for which no successful cure has been found. These patients lack an effective T cell immune response capable of activating MOi, and antimonial treatment only achieves transitory remission [2, 3 and 4].

Murine models infected with *L. major* have shown that both the innate and acquired immune responses are necessary for parasite clearance. Cells such as MO, dendritic cells, NK cells, CD8 and CD4 T lymphocytes; cytokines such as interleukin (IL)-12 and interferon gamma (IFNγ), pattern recognition receptors such as Toll like receptors (TLRs) [6], [7], [8] and effector molecules such as nitric oxide (NO) and superoxide anion (O_2_^-^) [9] have been reported to mediate protection, both in mouse models and in humans. It has also been proposed that CD8 T cells play a crucial role in infection clearance, although the role of CD8 cytotoxicity in disease control has not been elucidated. Elevated numbers of CD8 have been reported in blood and lesions of patients infected with *L. major* and *L. mexicana* and their protective role has been associated with IFNγ production [10]. Additionally, we have previously reported that the number of CD8 is importantly reduced in lesions of DCL patients infected with *L. mexicana*, as compared to LCL patients [11]. Thus, a comparative analysis of the overall immune effector functions of CD8 from LCL and DCL patients would permit a more precise definition of the role played by these cells in the disease outcome, both by their cytokine production as well as by their cytotoxicity. We here report a functional analysis of CD8 isolated from peripheral blood of LCL and DCL patients infected with *Leishmania mexicana*. Our results show significant differences between CD8 of both groups: while CD8 from LCL patients produce high levels of IFNγ and show cytotoxicity against autologous MOi, the CD8 from DCL patients show a diminished response both in cytokine production as well in *Leishmania*-specific cytotoxicity. Thereafter we analyzed if the differential cytotoxicity observed *in vitro* also correlated with the number of apoptotic cells in lesions of both groups of patients and found that DCL patients have significantly less TUNEL^+^ cells than LCL patients. The diminished CD8 response in DCL patients resembled the cellular “exhaustion” reported for CD8 in other chronic diseases [12], [13], [14], [15], where CD8-effector capacity could be restored by different mechanisms including TLR signaling [16], [17], [18]. Since TLR2 signaling in CD8 enhanced proliferation and survival *in vitro* [19], we analyzed whether TLR2 stimulation of CD8 from DCL patients could also restore their functional capacity. We therefore stimulated CD8 from DCL patients with the TLR2-specific agonist Pam3Cys and with *Leishmania mexicana* LPG, which has been shown to be a TLR2 ligand capable of activating human peripheral blood mononuclear cells and NK cells to produce Th1-promoting cytokines [6], [8]. We show that stimulation with TLR2-specific agonists Pam3CyS or with *Leishmania* LPG can restore the effector functions of CD8 from DCL patients, including IFNγ production, antigen specific cellular proliferation and cytotoxicity against MOi. In addition to restoring these functions, TLR2-stimulated CD8 cells showed a reduction in PD-1 expression, a molecule frequently present in cellular exhaustion. This phenomenon had previously been described with the TLR9 ligand CpG ODN in mice [18].

## Materials and Methods

### Patients

Human experimentation guidelines of the Mexican Health authorities were strictly followed. The study was reviewed and approved by the Ethics Research Committee of the Medical Faculty of UNAM. Written informed consent was required for all patients. Ten individuals with LCL (4 females and 6 males, mean age = 34.6±13.9) and four with DCL (1 female and 3 males, mean age = 46±12.9) from La Chontalpa – Tabasco State (except one DCL), an endemic area in southeastern Mexico, were analyzed. Patients were diagnosed by clinical criteria, parasite presence in lesions, and immunoreactivity to the Montenegro skin test. LCL patients showed skin ulcers containing few parasites and all were positive to the Montenegro test. In contrast, DCL patients had multiple non-ulcerative nodules, harboring an intense parasite load and all were negative in the Montenegro test.

Peripheral blood for *in vitro* experiments was collected in tubes with EDTA (BD Biosciences). Skin biopsy specimens were taken from the lesions with a 4 mm biopsy punch (Stiefel Laboratories, Inc., Coral Gables, FL) after local anesthesia (2% xylocaine). The biopsy specimens were embedded in OCT compound (Miles Scientific, Napperville, IL), snap frozen and stored in liquid nitrogen until examined. All patients received antimonial therapy after sample collection.

### Immunohistochemistry

Frozen sections were cut with a cryostat and air-dried overnight before the immunostaining procedure. Apoptotic cells in lesions of 7 LCL and 5 DCL patients were detected by the TUNEL method (In Situ Cell Death Detection Kit, POD, Roche). Briefly, cryostat sections (4 µm) were thawed onto coated slides, fixed in paraformaldehyde 4% in PBS pH 7.4 for 20 min, washed 30 min with PBS and incubated with permeabilization solution for 2 min on ice. Slides were rinsed twice with PBS and 50 µl TUNEL reaction mixture were added to the tissue sections and incubated for 60 min at 37°C in a humidified atmosphere in the dark. Slides were washed 3 times with PBS and incubated with 50 µl of Converter-POD (Anti-fluorescein antibody conjugated with horse-radish peroxidase) in a humidified chamber for 30 min at 37°C. Slides were washed 3 times with PBS and incubated with 50 µl of DAB substrate for 10 min at 25°C and washed 3 times with PBS. Then, samples were mounted under glass coverslips and analyzed under light microscope. Double staining with anti-CD8 antibodies was carried out in six samples of LCL patients as follows: after TUNEL staining, slides were washed with 100 mM Tris-HCl, pH 7.4, 150 mM NaCl. The samples were blocked with Blotto (a solution containing 5% skim milk powder and 0.1% Tween 20 in PBS, pH 7.4) for 30 min at RT. The samples were incubated with primary mouse anti-CD68 (1∶100; Dako: Dako Corp., Santa Barbara, CA) during 1 h in a humid chamber at RT. Five-minute washes with 10 mM Tris-HCl, pH 7.4, 150 mM NaCl were followed by 1 h incubation with biotin-conjugated second-step antibody (goat anti-mouse IgG) at a dilution of 1∶50 and with the preformed streptavidin-biotin alkaline phosphatase complex (Dako) for 1 h at RT. The presence of alkaline phosphatase was evidenced by incubation in AP substrate solution containing 1 mM levamisole for 30 min. Then, slides were washed twice in distilled water. Counterstaining was performed with H&E (Sigma Chemical). The apoptotic cells were identified in a light brown color, whereas MO showed red staining.

### *Leishmania mexicana* culture

*Leishmania mexicana* (MHOM/MX/92/UADY/68) promastigotes were grown in RPMI-1640 medium (Life Technologies Laboratories, Gaithersburg, MA, USA) supplemented with 5% heat-inactivated FBS at 28°C. Parasites were sub-cultured every 4 to 5 days and grown to a density of 1×10^6^/ml. Promastigotes were harvested from stationary-phase cultures, centrifuged at 3500 rpm for 10 min, washed three times in PBS, and finally counted after immobilization with glycerol.

### CD8 and MO purification

Peripheral blood mononuclear cells were separated by Ficoll-Hypaque gradient (Sigma) and CD8 were isolated by magnetic cell sorting system as described by manufacture' instructions (Miltenyi Biotec, Bergisch Gladbach, Germany). Briefly, 1×10^7^ PBMC was suspended in 40 µl PBS containing 10 µl of anti-CD8 microbeads and incubated for 30 min at 4°C. The cells were washed with PBS and centrifuged at 1500 rpm for 10 min. They were then passed through the LS separation columns (Miltenyi) placed in magnetic field. The positive fraction was cultured in RPMI/FBS 10%. The negative fraction was processed again in order to purify MO (1×10^7^ cells in 40 µl of PBS with 10 µl of anti-CD14 microbeads). MO were cultured in RPMI/FBS 10%. Both CD8 and MO were maintained at 37°C in a humidified atmosphere containing 5% CO_2_ incubator for 12 h before stimulus in order to reach a resting condition.

### MO infection

1×10^6^ MO were co-incubated with 1×10^7^ *L. mexicana* promastigotes for 3 h at 28°C in 1 ml RPMI. Afterwards, cells were cultured for additional 18 h at 37°C. Non-ingested promastigotes were washed away with RPMI.

### Cytotoxicity assay

The cytotoxicity of CD8 cells on MOi was analyzed by two methods: ^51^Cr-release and Flow cytometry. The optimal CD8/MOi ratio was established as 10/1.

#### ^51^Cr-release assay

The assay was performed as previously described [20]. Briefly, MO or MOi were labeled with 100 µCi of ^51^Cr (Perkin Elmer) for 1 h at 37°C, 5% CO_2_. The labeled cells were washed twice in RPMI, suspended in RPMI and 1×10^6^ CD8 were co-incubated with autologous 1×10^5^ MOi. Co-cultures were set up in 24 well flat bottom plates (Linbro, Aliso Viejo, CA) and plates were incubated in triplicate for 4 h at 37°C in 5% CO_2_. Controls included MOi incubated in medium alone for spontaneous release and MOi incubated in 5% (v/v) Triton X- 100 (Sigma, St. Louis, MO) in PBS for maximum release. Radioactivity was measured by Wallac Wizard1470 Automatic Gamma Counter. The cytotoxicity percentage was calculated using the formula: % cytotoxicity  =  (sample cpm – spontaneous cpm) / (maximal cpm – spontaneous cpm) ×100%.

#### Flow cytometric assay

The assay was performed as previously described [21]. Briefly, 1×10^6^ CD8 were mixed with autologous 1×10^5^ MOi or MO in 24 well clear flat bottom ultra low attachment plates and incubated at 37°C with 5% CO_2_ for 4 h. After incubation, 5 µl of Ab anti-CD14 PE (BD: Becton Dickinson Biosciences, San Jose CA) were added for 15 min at 4°C. Afterwards, 10 µl of a 5 µg/ml solution of 7-AAD (Sigma) diluted in Annexin Binding buffer 1× from 10× concentrate (BD), and 5 µl of annexin V-FITC (BD) were added to the cell suspension for 10 min on ice. Samples were analyzed on a FACSCanto II flow cytometer (BD). A minimum of 20 000 events were collected and analyzed with DIVA software from the instrument manufacturer. The cytotoxicity index (C.I). was calculated using the formula: C.I.  =  {(CD14^+^ annexin V^+^) of CD8 incubated with MOi} – {(CD14^+^ annexin V^+^) of CD8 incubated with MO}.

In a parallel experiment, the expressions of FasL and Granzyme B were evaluated using Ab anti-FasL – PE (BD) and anti-granzyme – Alexa Fluor 647 (BD). The supernatant was harvested for IFNγ detection by means of ELISA test.

### Proliferation assay

The proliferation of CD8 in response to autologous MOi was analyzed as previously described [22]. Briefly, PBMC were suspended in RPMI with CFDA (Sigma) [5 µM] during 10 min at 37°C and washed twice in RPMI. Cells were cultured at 1×10^6^ per ml and 2×10^5^ MOi were added. MO or Con A [5 µg/ml] (Sigma) were used as controls. The cells were co- cultured for 7 days at 37°C. Cells were harvested and incubated in the presence of anti-CD3 CyChrome and anti-CD8 PE (BD). Stained samples were fixed with 2% paraformaldehyde in PBS, and analyzed by multicolor flow cytometry immediately after the end of the incubation period. The percentage of CD3^+^CD8^+^CFDA^low^ was recorded.

### IFNγ production

The effect that MOi had on the production of IFNγ by CD8 was analyzed as follows. 1×10^6^ PBMC were co-incubated with 2×10^5^ MOi or MO during 18 h after which 1 µl GolgiPlug (BD) was added and incubated for additional 5 h. Cells were harvested and incubated in the presence of anti-CD8 PE (BD). They were washed twice with PBS. Cells were suspended in Citofix/Citoperm (BD) and washed with Perm/Wash. Then, cells were incubated with anti- IFNγ PE-Cy7 (BD) and washed twice on Perm/Wash. Stained samples were suspended in PBS and analyzed by multicolor flow cytometry.

IFNγ production was also analyzed in the supernatants of the cytotoxicity experiments using standard ELISA assays. In brief, 96-well microtitre plates (Costar, Corning, NY) were coated with un-conjugated anti-IFNγ (clone NIB42; 6 µg/ml; BD) capture antibody in 100 mM Na2HPO4, pH 9.0 during 12 h at 4°C, and blocked with PBS 0.1N NaOH, 0.5% casein, pH 7.4. Supernatants and hIFNγ recombinant standard (R&D Systems, PR) were incubated in RPMI/FBS 10% during 2 h at RT. hIFNγ was detected using biotinylated mouse Ab anti-hIFNγ (clone 4S.B3, 0.5 µg/ml; BD) in 1% BSA, 0.05% Tween 20 using streptavidine labeled with alkaline phosphatase (Life Technologies) and p-nitrophenil phosphate (4 mg/ml, Life Technologies) as substrate. Absorbance was read at 405 nm and IFNγ concentration was evaluated in the hIFNγ recombinant standard curve. IFNγ concentration in every sample was calculated by lineal regression using mean absorbance (average of three lectures by sample). Detection limit was of ∼15 pg/ml.

### Stimulation with TLR2 ligands

Purified *Leishmania mexicana* LPG was obtained as previously described (6). Commercial Pam3Cys-Ser-(Lys) 4 (PAM) was used as control (EMC Microcollections GmbH, Tübingen, Germany). Purified CD8 (1×10^6^) of DCL patients were stimulated with LPG 10 µg/ml or PAM 2 µM during 24 h. Cells were harvested, washed twice, and used in cytotoxic assays. For proliferation and IFNγ production assays, 1×10^5^ pre-stimulated purified CD8 were co-incubated with autologous 9×10^5^ PBMC and experiments were carried out as described above. Additionally, PD-1 expression was analyzed in non-stimulated and stimulated CD8 stained with anti-CD8 PE, anti-CD3 CyChrome and anti-PD-1 FITC.

Data are expressed as mean ± SEM and were tested using Mann–Whitney U-test or Kruskall-Wallis test. A value of *p*<0.05 was considered statistically significant.

## Results

### CD8 cytotoxic against autologous MOi

**Figure 1:**
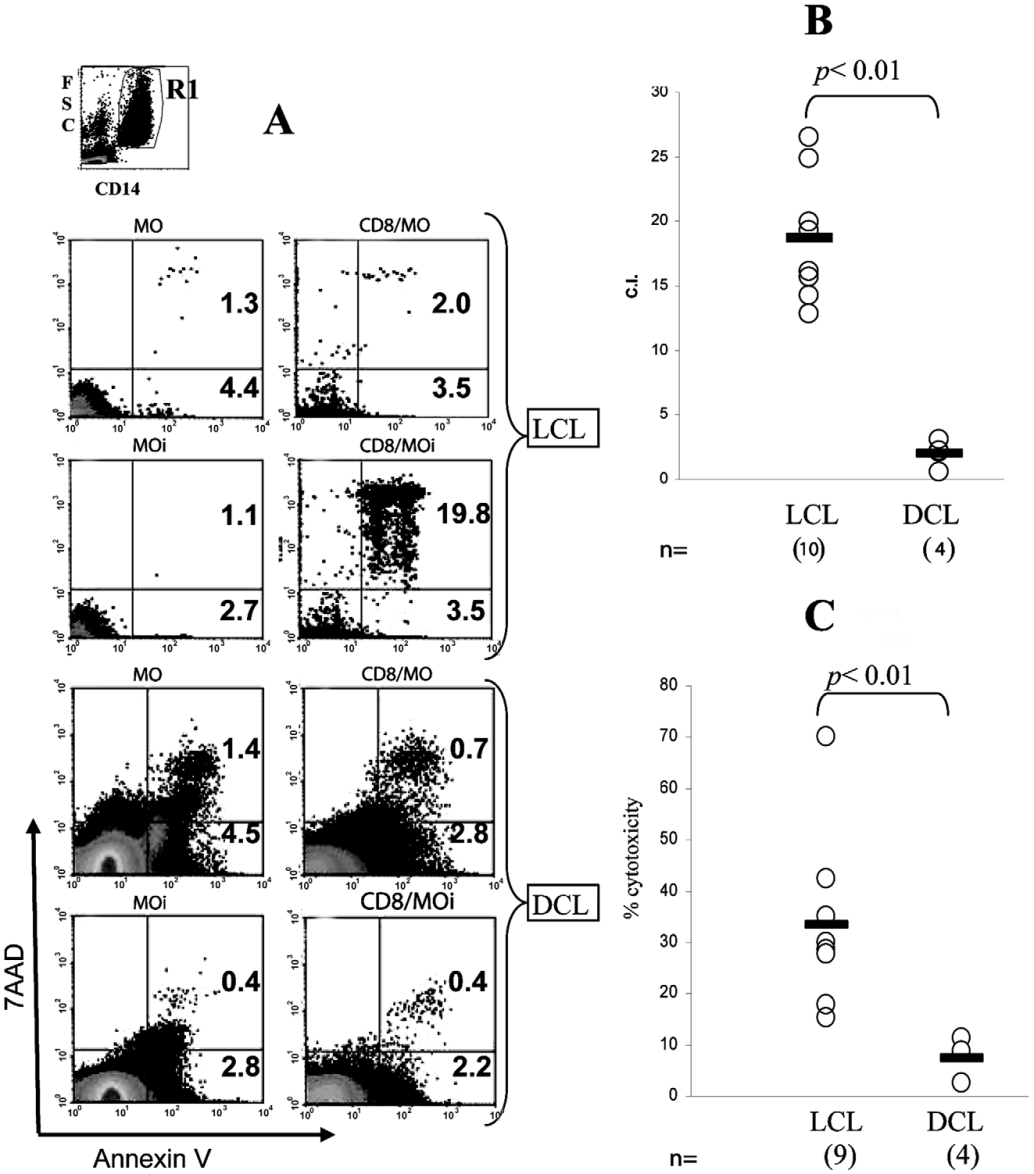
Cytotoxicity assay of CD8 against autologous *Leishmania-*infected macrophages (MOi). A) Culture dotplots (annexin V *vs.* 7AAD) from MO, MOi, CD8/MO, and CD8/MOi of LCL and DCL patients. In top, the R1 region corresponds to the CD14^+^ cells analyzed. The percentage of annexin V^+^ cells are shown. B) Analysis of flow cytometry assay. Cytotoxicity index (C.I.) was calculated from 10 LCL and 4 DCL patients. C) Analysis of Cr^51^ release assay. The % cytotoxicity was calculated from 9 LCL and 4 DCL patients. The bars show the mean of C.I. and % cytotoxicity. *p* was obtained by the Mann-Whitney U test.

In our previous report about cellular infiltrate in lesions of DCL patients we described that the number of CD8 is importantly reduced as compared to LCL patients [11]. The reduction in the number of CD8 cells in lesions of DCL patients led us to analyze if their cytotoxic effector function was also altered. Thus, we analyzed the cytotoxic capacity of CD8 of LCL and DCL patients against autologous MOi. The cytotoxic capacity was measured by two methods: flow cytometry and ^51^Cr radiolabeling (Figure 1). Prior to the cytotoxic experiments, the optimal CD8: MOi ratio had been established as 10∶1 (data not shown). The cytometry assay revealed that patients with LCL had a C.I. of 19.8 as compared to DCL patients, which showed a C.I. of 0.4, as evidenced by double positive MOi dot blots (Figure 1A and 1B). Additionally, ^51^Cr radiolabeling showed that the mean percentage of cytotoxicity in LCL patients was 18% as compared to 3% en DCL patients (Figure 1C). Thus, both methods showed a significantly higher cytotoxicity of CD8 cells from LCL patients, as compared to DCL patients, where cytotoxicity was almost null (*p*<0.01). In order to rule out the possibility that CD8 death could be responsible for the low cytotoxicity in DCL patients, we analyzed CD8 expression of annexin V by flow cytometry assay and found that CD8 do not undergo apoptosis when they are co-incubated with MOi (data not shown).

### CD8 cytotoxic mechanism against autologous MOi in LCL

**Figure 2:**
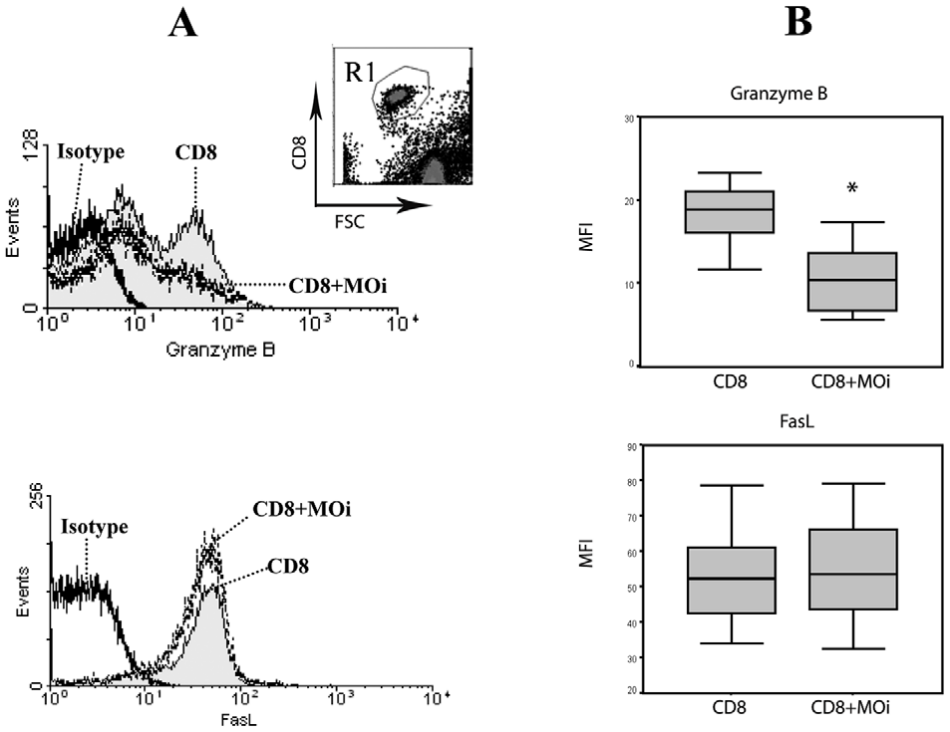
Cytotoxic mechanism analysis. A) LCL patient CD8 were labeled for granzyme B or FasL in CD8 alone or co-cultured with MOi (CD8+MOi) 1∶1 for 4 h. R1 was analyzed from the CD8 vs FSC dotplot (top). Data are representative of 10 LCL patients. B) Mean fluorescence intensity (MFI) for Granzyme B and FasL expression were analyzed in 10 LCL patients. T-student test was carried out between CD8 and CD8+MOi (*p*<0.01).

CD8 T lymphocytes can exert cytotoxicity by two mechanisms: granule exocytosis and death ligands. Granzyme B and FasL are prominent executor molecules of these pathways [23]. To determine the mechanism by which CD8 kill MOi, we measured the expression of granzyme B and FasL, before and after cytotoxic challenge with autologous MOi from LCL patients, which were the only ones that had exhibited cytotoxic capacity. These assays however were carried out in a CD8:MOi ratio of 1∶1 since this permitted better FACS analysis of protein expression in CD8 T cells (Figure 2). Whereas FasL expression remained unchanged, granzyme B expression decreased significantly (*p*<0.01) (Figure 2B) after the cytotoxic challenge, which suggests that cytotoxicity is mediated by cytotoxic granules.

### Specific CD8 proliferation

**Figure 3:**
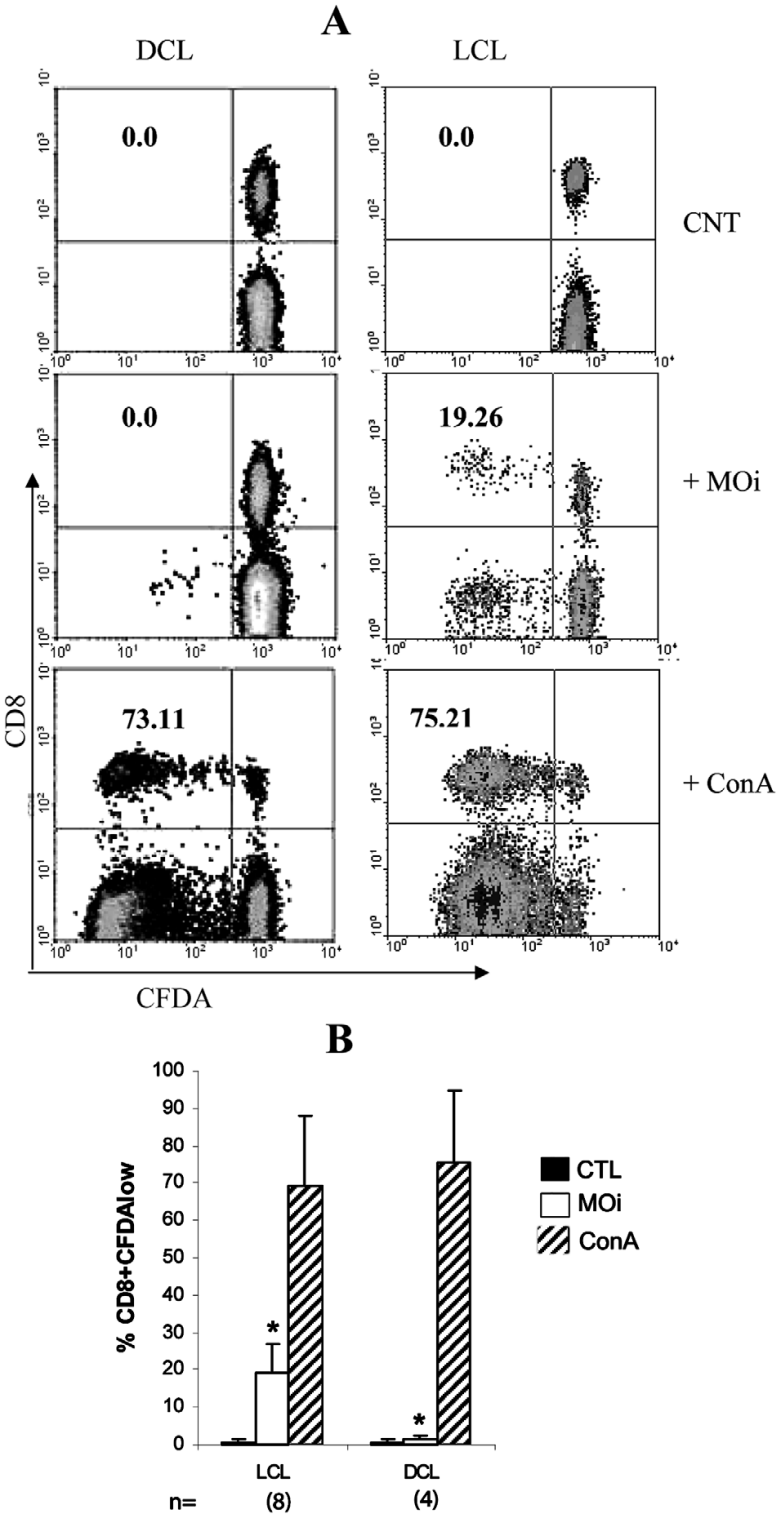
Lymphocyte proliferation in LCL and DCL patients. A) CFDA-labeled PBMC were incubated with PBS (CNT), MOi or Con A [5 µM] for seven days. CFDA^low^ cell percentages of total CD8 are shown. Representative dotplots from 8 LCL and 4 DCL patients are shown. B). Proliferation analysis of CD8 from 8 LCL and 4 DCL patients, stimulated with PBS (black bars), MOi (white bars) and Con A (striped bars) are shown (*p*<0.01).

As CD8 from DCL patients had shown a different cytotoxic capacity with regard to LCL patients, we investigated other functions that could be altered in the cells of both groups of patients, such as specific proliferation of cells in contact with *Leishmania* antigens. CD8 proliferation was evaluated in PBMC cultures incubated with MOi. After 7 days of culture, a CD8^+^CFDA^low^ group was detected in LCL patients, formed by cells that proliferated as a response to the stimulus of MOi (Figure 3A). This group was not found in DCL patients, however, a CD8^-^CFDA^low^ group mainly consisting of CD3^+^CD4^+^ cells was detected (data not shown). The proliferation of CD8 was only evidenced when the cells were stimulated with MOi and the difference between LCL and DCL patients was statistically significant (% CD8^+^CFDA^low^ MOi LCL  = 18.37+/−7.1 vs DCL  = 2.3+/−1.5) (*p*<0.01) (Figure 3B). Stimulation with Con A, as positive control, did not show differences in CD8 proliferation between LCL and DCL patients. This shows that the lack of response in CD8 of DCL patients is specific towards autologous MOi with *Leishmania*.

### IFNγ production by CD8

**Figure 4:**
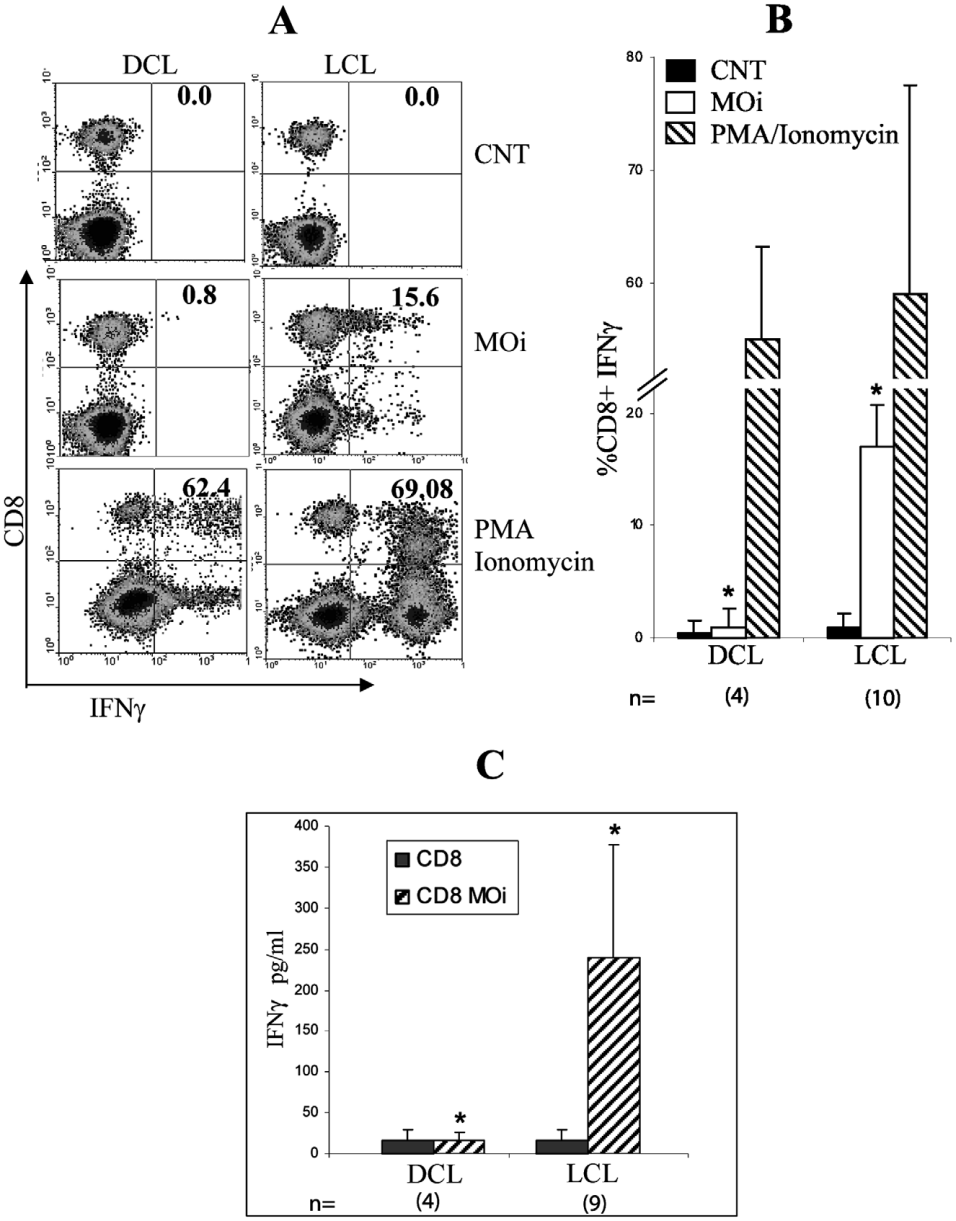
IFNγ production by CD8 from LCL and DCL patients. A) PBMC were non-stimulated (CNT), stimulated with PMA-Ionomycin during 4 h or with MOi during 24 h. Density diagrams of IFNγ vs. CD8 from 10 LCL and 4 DCL patients are shown. B) CD8^+^IFNγ^+^ percentage analysis from 10 LCL and 4 DCL patients are shown. C) ELISA results of IFNγ production in supernatants of the co-incubation CD8-MOi from 9 LCL and 4 DCL patients are shown (*p*<0.01).

Another reported function of CD8 T cells in leishmaniasis is IFNγ production [10]. To examine if there are differences in the cytokine secretion of between CD8 of LCL and DCL patients when they are stimulated with autologous MOi, a 24-h *in vitro* assay was performed after which lymphocytes were labeled for intracellular IFNγ. Only CD8 LCL patients showed response to MOi (%CD8^+^IFNγ^+^ DCL = 1.8+/−2.5 vs LCL  = 17.5+/−3.9) (*p*<0.01) (Figure 4A and 4B). This group of CD8^+^IFNγ^+^ cells was not detectable in DCL patients. None of the patients showed CD8^+^IL4^+^ cells (data not shown). IFNγ was also measured in the supernatants of flow cytometric cytotoxic assays by ELISA. The results show that IFNγ was only detected in LCL patients (IFNγ CD8+MOi DCL  = 20 pg/ml +/−4 vs LCL  = 241.8pg/ml +/−137.1) (*p*<0.01) (Figure 4C). The lack of IFNγ production by CD8 of DCL was specific towards *Leishmania* antigens since an appropriate response was found to the non-specific mitotic stimulus PMA/Ionomycin (Figure 4A and 4B).

### Apoptosis in tissues

**Figure 5:**
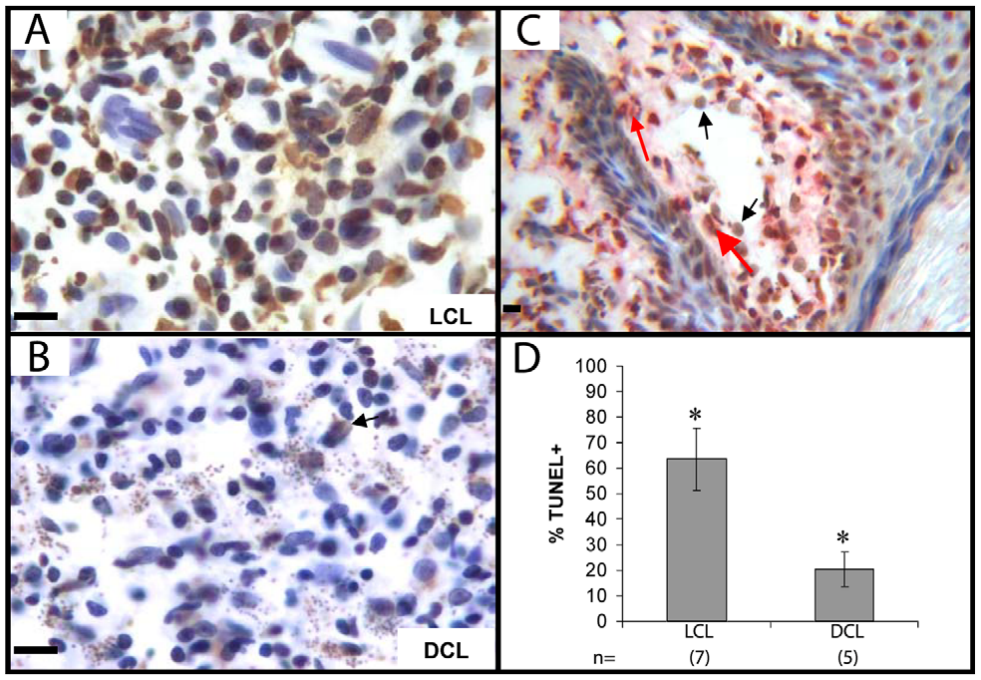
Apoptosis in LCL and DCL patient lesions. Frozen tissue sections of LCL (A) and DCL (B) were stained with TUNEL. Two representative photographs are shown. C) Double staining of LCL patient tissue: TUNEL in peroxidase (brown), CD68 in phosphatase (red). Black arrows show TUNEL^+^ cells, red arrows show TUNEL^+^ CD68^+^ cells. D). Positive percentage analysis for TUNEL of 7 LCL and 5 DCL patients is shown. Three fields were counted, 200 cells per field (*p*<0.05). Bar  = 20 µm.

As *in vitro* CD8 cytotoxic assays showed an impaired function of DCL cells as opposed to the robust response found in LCL patients (Figure 1), we examined if biopsies of skin lesions of both groups of patients showed any evidence of the different cytotoxicity between both groups of patients We analyzed apoptotic cells through TUNEL staining in skin biopsies of LCL and DCL patients (Figure 5A and B). The stain revealed a high proportion of apoptotic cells in tissues of 7 LCL patients (Figure 5A), which was significantly higher as compared to 5 DCL patients (% TUNEL^+^ LCL  = 63.4% +/−12.08 vs DCL  = 20.9% +/−7.43) (*p*<0.01) (Figure 5B and 5D). Double staining in LCL patients showed that a large number of apoptotic cells were MO (Figure 5C).

### Evaluation of the TLR2 stimulus in CD8 of DCL patient

**Figure 6:**
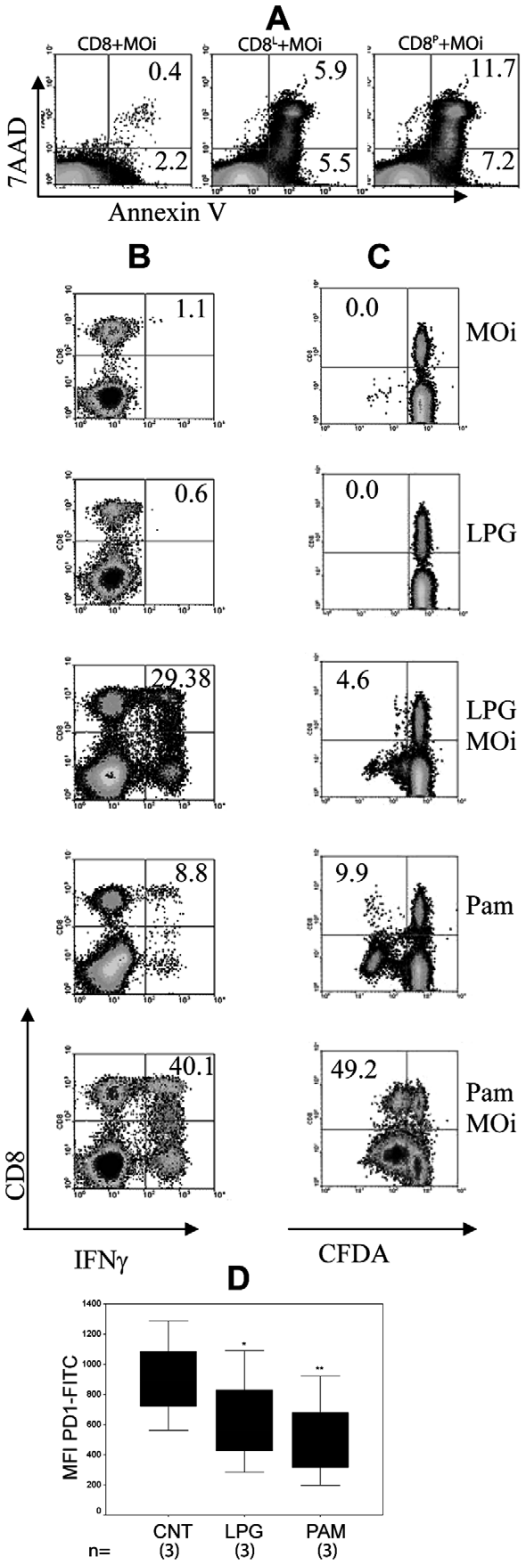
Effector functions of CD8 from DCL patients were restored by pre-stimulation with TLR2 ligands. CD8 from DCL peripheral blood were purified and incubated in *L. mexicana* LPG [10 µg/ml] (CD8^L^) or Pam3Cys [2 µg/ml] (CD8^P^) for 24 h and washed twice. CD8 were analyzed for: A) Cytotoxicity, B) IFNγ production and C) Proliferation. One representative of 3 DCL patients is shown. D) Analysis of mean fluorescence intensity (MFI) for PD-1 expression on non-stimulated (CNT), LPG or Pam3Cys-stimulated CD8 from 3 DCL patients is shown (*p = 0.02, **p = 0.005).

As CD8 from DCL patients had shown deficiencies in *Leishmania*-specific cellular effector functions, we analyze if this apparent “exhaustion” could be reversed by TLR2 stimulation, as had been previously shown for other chronic diseases. We examined cytotoxicity, antigen-specific proliferation and IFNγ production of CD8 from DCL patients, which had previously been stimulated with LPG or Pam3Cys for 24 h. We observed a recovery of the CD8 effector functions, including cytotoxicity, IFNγ production and proliferation (Figure 6A, B and C). The stimulus with Pam3Cys induced higher levels in IFNγ production as compared to LPG. The response was highest when CD8 were prestimulated with TLR2 ligands and challenged with MOi (Figure 6B). Proliferation was also induced in CD8 cells although they were not the only ones that responded to the stimulus, since a population of CD8^-^ lymphocytes also proliferated (Figure 6C). Again, Pam3Cys plus MOi could induce the highest response. Since exhausted T cells have been shown to have increased expression of programmed death-1 (PD-1) molecules, which could be modified by TLR-9 ligand, we analyzed if PD-1 expression in CD8 cells of DCL patients could be modified by TLR2 agonists. We found that PD-1 expression in CD8^+^ of DCL patients was significantly reduced after stimulation with both TLR2 agonists, LPG (*p* = 0.02) and Pam3Cys (*p* = 0.005) (Figure 6D).

## Discussion

The protective immune response against *Leishmania*, amply studied in mice, is mediated by macrophage-activating cytokines, such as IFNγ and IL-12 [5]. MO activation involves iNOS expression and NO production, the latter being the most important leishmanicidal agent [24]. In humans, IFNγ production and iNOS expression have also been directly associated with the resolution of infection. Additionally, CD8 have been shown to play an important role during *Leishmania* infection, both in humans as well as in the mouse model. During the acute phase of the disease, large numbers of CD8 have been described in the lesions as well as in the peripheral blood of patients and they have also been observed during the healing process [10]. Yet thus far, the important role of CD8 in leishmaniasis has been related to their IFNγ production [25], [26] and little is known of the protective response mediated by the cytotoxicity exerted by these cells [27]. It is additionally not clear, if these cytotoxic cells relate with disease progression in patients with diffuse cutaneous leishmaniasis.

Previous studies of our group have shown that lesions of DCL patients infected with *L. mexicana* harbor low numbers of CD8, as compared to patients with LCL. Interestingly, the leishmanicidal treatment of the DCL patients not only reduced the parasite load but also led to an increase in CD8 during the healing process of the skin lesions of these DCL patients [11]. To date, the mechanism responsible for the reduced presence of CD8 in lesions of DCL patients remains unclear. The marked response of CD8 towards *Leishmania* parasites led us to analyze possible functional differences between these cells from patients with LCL and DCL. We therefore compared *in vitro* antigen-specific proliferation, IFNγ production and cytotoxicity of CD8 purified from PBMC of both groups of patients against autologous macrophages infected with *Leishmania mexicana.* Additionally we analyzed tissue biopsies from both groups of patients for evidence of cytotoxicity associated with apoptotic cells in the lesions.

Our data demonstrate that CD8 from DCL patients show a significant reduction in their effector response when coincubated *in vitro* with autologous macrophages infected with *Leishmania mexicana*, as compared to patients with LCL. This diminished cytotoxic response was also evidenced in lesions of both groups of patients, since DCL patients showed significantly less apoptotic cells as compared to LCL patients (Figure 5). Although not much is known of the overall effector mechanisms of CD8 T cells in human leishmaniasis, results obtained in the present work show that both IFNγ production, as well as cytotoxicity against *Leishmania-*infected macrophages are hampered in DCL cells. It is therefore feasible to speculate that CD8 cells play an important role in the protective response of LCL patients against the infection with *Leishmania mexicana*, based on our *in vitro* results, together with the large number of apoptotic cells in the tissue lesions of these patients. Although data on the expression of apoptosis in skin lesions are scarce, our work is in accordance with the literature, where apoptotic CD4 and CD8 T lymphocytes have been described in lesions of LCL patients infected with *L. braziliensis*, albeit cell death was attributed to hypersensitivity towards *Leishmania* antigens, leading to activation-induced cell death [28].

MO cell death in tissues could be due to *Leishmania* infection and/or be a consequence of CD8 cytotoxic activity against infected cells. The former mechanism is unlikely, since it has been demonstrated that *Leishmania* can inhibit apoptotic mechanisms in phagocytic cells [29], [30], [31], [32]. The possible benefits derived from cytotoxic CD8-induced apoptosis of the host cells infected with *Leishmania* could be dual: the elimination of the parasite and the expansion of the specific immune response by providing novel parasite antigens [10], as has described for *M. tuberculosis* [33].

Controversy remains regarding the route of activation of CD8 in leishmaniasis, since these cells require antigen presentation through MHC class I to become activated. It is not known how *Leishmania* antigens escape from the parasitophorous vacuole of phagocytic cells into the cytosol to be degraded and transported into the endoplasmic reticulum to be bound to the MHC I molecule. One of the possible mechanisms described in dendritic cells and macrophages is through cross-presentation and involves the chaperone protein Sec61. Another mechanism of cross-presentation of exogenous antigens could be through phagocytosis of apoptotic bodies of infected cells by dendritic cells, which aids the immune response by providing novel parasite antigens [10].

Good prognosis in patients with cutaneous leishmaniasis, infected with *L. major*, has been associated with the expression of granzyme B in tissue lesions [34]. In the present work, granzyme B was also associated with the cytotoxic activity of CD8 of LCL patients against MOi (Figure 2).

Since the presence of CD8 in the lesion has also been correlated with recovery of *Leishmania-*infected patients [11], it is feasible that lack of cytotoxicity of CD8 against MOi (Figure 1) can lead to uncontrolled parasite spread in DCL patients. However the functional efficacy of CD8 cells from patient tissues cannot be comparatively analyzed with those from peripheral blood due to the small size of lesions in LCL patients, which are often no larger than 2 cm of diameter and therefore limit the amount of lymphocytes available for functional experiments. Although we cannot rule out functional differences between CD8 T cells from lesions and those from peripheral blood, data in the literature have suggested that immunological responses *in vitro* of peripheral blood mononuclear cells closely mimic the immune response that occurs in the whole organism [35], [36].

One of the possible mechanisms underlying the reduced effector capacity CD8 of DCL patients could be a functional “exhaustion” of these cells induced by a suppressive environment and antigen persistence, as has recently been described [13]. Despite the small number of DCL patients available for this study, due to the low incidence of this form of the disease in Mexico, we found that CD8 of these patients showed certain functional and phenotypic characteristics that resemble the exhaustion condition described in some chronic viral infections [12]. “CD8 cell exhaustion” was initially described in chronic LCMV infection in mice, in which virus-specific CD8 persist, but lack effector functions [37]. In this viral infection, CD8 clones are initially generated, but cytotoxicity and proliferation are lost at an early stage, while IFNγ persists for a longer period [38]. Similar types of dysfunctions have been described in human chronic infections and during cancer [12], which suggests that chronic antigen stimulation alone suffices to drive CD8 into exhaustion [13]. Recently in a murine model of *L. donovani* infection, it has been describes that parasites initially induce CD8 to divide and produce IFNγ. However, CD8 rapidly lose their effector functions and die as the infection progresses [16]. Our present results suggest that the same phenomenon could be occurring in DCL patients, which have a chronic intracellular infection of more than 20 years evolution. These patients do not present delayed cellular hypersensitivity toward specific *Leishmania* antigens, as evidenced by their negative Montenegro skin test, nor do they present a cell-mediated immunity towards the parasite [1] (Figures. 1, 3 and 4). It must be noted that the lack of lympho-proliferative response and the lack of IFNγ production of CD8 from DCL patients was specific against *Leishmania* antigens, since these cells preserved their capacity to respond to mitogens such as Con A and PMA/Ionomycin (Figures. 3 and 4). These results suggested that a functional exhaustion towards *Leishmania mexicana* could be present in CD8 of DCL patients. “Exhausted” cells have been shown to express higher levels of PD-1, among other inhibitors, and essays of restoration of these exhausted T-lymphocytes have focused on the use of anti-PD-L1 and PD-L2 antibodies, which prevent binding of T-cell PD-1 to the antigen-presenting cell (APC) ligands PD-L1 and PD-L2 [16], [17], [38]. In these studies, TCR and CD28 signals sufficed to rescue CD8 in exhaustion, once PD-1 activity was blocked [17].

Recently, Wong and co-workers [18] demonstrated that peptide vaccination in the presence of CpG ODN (TLR9 ligand) reduced expression of PD-1 in mice. In addition to TLR9, another innate receptor that has recently been described in CD8 is TLR2, which triggers cellular activation similar to that of CD28 [39]. Therefore, we hypothesized that TLR2 stimulation could reduce PD-1 expression and restore CD8 functional activity in DCL patients against MOi. Since we had previously shown that LPG, the most abundant surface molecule expressed on the *Leishmania*, is a TLR2 ligand capable of inducing IFNγ production in human NK cells [6], we therefore stimulated CD8 from DCL patients with LPG and also with Pam3Cys, a recognized specific TLR2 ligands (Figure 6A, B and C). We found that this preactivation could increase CD8 cytotoxicity against MOi. Also, proliferation and IFNγ-producing CD8 could be detected when they were pre-stimulated with LPG or Pam3Cys.

Due to a lack of knowledge of *Leishmania*-specific CD8 T cell epitopes, antigen-specific CD8 responses in CL have not been studied and therefore phenotypic characterization of possible CD8 exhaustion has not been feasible.

Although CD8 exhaustion has not been reported in patients with leishmaniasis, our results on “functional exhaustion” is in accordance with the literature since Joshi and coworkers could demonstrate that *L. donovani* limits CD8 expansion and induces functional exhaustion in an experimental model [16] which was associated with increased PD-1 expression by *Leishmania*-specific CD8. Our analysis also showed that PD-1 expression on CD8 of DCL seems to be associated with functional exhaustion. We were able to show that PD-1 expression on CD8 from DCL patients could be reduced by stimulation with TLR2 ligands and that the reduction correlated with functional restoration (Figure 6D).

These results suggest that stimulation by TLR2 could represent a pathway hierarchically higher than PD-1. The TLR2 receptors are constitutively expressed in memory T lymphocytes, both in CD4 and CD8, and their activation leads to a co-stimulating signal that induces cytokine production and proliferation [39]. Moreover, TLR2 - MyD88 signaling is a critical pathway in CD8 clonal expansion and memory formation *in vivo* [19]. Additionally, TLR signaling influences Treg function, in particular, Pam3Cys has been shown to transiently reduce the expression of FoxP3^+^ in Treg cells and to suppress their activity [40]. Thus, TLR2 stimulation is not only limited to innate immune responses, but also regulates the adaptive immune response.

In conclusion, we here show that CD8 of DCL patients lack a cytotoxic activity against autologous macrophages infected with *Leishmania mexicana* and they present functional and phenotypic characteristics of exhausted cells, possibly as a consequence of chronic intracellular infection by *Leishmania mexicana*. We additionally present the first evidence in the literature that TLR2 stimulation can restore their effector functions. It remains to be determined if the exhausted condition of CD8 towards parasite antigens is a cause or a consequence of the progressive infection of DCL patients by *Leishmania mexicana* and if TLR2 induces downregulation of PD-1 pathway. This finding not only broadens our knowledge of the pathogenesis of the disease but will allow the design of activators of CD8 from DCL patients, based on TLR2 stimulation of the immune response.
